# Application of the Combination of Soybean Lecithin and Whey Protein Concentrate 80 to Improve the Bile Salt and Acid Tolerance of Probiotics

**DOI:** 10.4014/jmb.2103.03017

**Published:** 2021-05-07

**Authors:** Xuelei Gou, Libo Zhang, Shiwei Zhao, Wanping Ma, Zibiao Yang

**Affiliations:** Yunnan Huangshi Lesson Dairy Industry Co., Ltd., Dali 671000, P.R. China

**Keywords:** *Lacticaseibacillus paracasei* L9, bile salt tolerance, acid tolerance, soybean lecithin, whey protein concentrate 80, response surface methodology

## Abstract

To improve the bile salt and acid tolerance of probiotics against gastrointestinal stresses, we investigated the effects of soybean lecithin and whey protein concentrate (WPC) 80 on the bile salt tolerance of *Lacticaseibacillus paracasei* L9 using a single-factor methodology, which was optimized using response surface methodology (RSM). The survival rate of *L. paracasei* L9 treated with 0.3% (w/v) bile salt for 2.5 h, and combined with soybean lecithin or WPC 80, was lower than 1%. After optimization, the survival rate of *L. paracasei* L9 incubated in 0.3% bile salt for 2.5 h reached 52.5% at a ratio of 0.74% soybean lecithin and 2.54% WPC 80. Moreover, this optimized method improved the survival rate of *L. paracasei* L9 in low pH condition and can be applied to other lactic acid bacteria (LAB) strains. Conclusively, the combination of soybean lecithin and WPC 80 significantly improved the bile salt and acid tolerance of LAB. Our study provides a novel approach for enhancing the gastrointestinal tolerance of LAB by combining food-derived components that have different properties.

## Introduction

In 2001, the Food and Agriculture Organization (FAO) and the World Health Organization (WHO) defined probiotics as “live microorganisms which when administered in adequate amounts confer a health benefit on the host.” Probiotics are the most effective and accessible tools for modulating gut microbiota and thereby altering human health and diseases. Presently, lactic acid bacteria (LAB) and *Bifidobacterium* are used as traditional and universal probiotics in supplements or fermented foods. Probiotics demonstrate a range of effects, and gut microbiome dysbiosis is associated with gastrointestinal, autoimmune, neurological, and metabolic diseases [1]. The clinical efficacy of probiotics in some diseases, such as antibiotic-associated diarrhea [2], *Clostridium difficile*-associated diarrhea [3], and irritable bowel syndrome and inflammatory bowel disease [4] has been determined by manipulating the microbiota with probiotics. Additionally, the therapeutic success in some cancers is also associated with the diversity and composition of the gut microbiome [5].

Apart from the various health-promoting benefits, the most important characteristic of probiotics is that viable microbiota can pass through the acidic and high bile salt environments in the stomach and duodenum to exert their function [6, 7]. During production and consumption, probiotics face various stresses such as acid, bile salt, osmotic pressure, temperature and oxygen. To confer health benefits to the host, viable cells should reach and colonize the lower gastrointestinal tract. Therefore, probiotics should be resistant to the deleterious effects of gastric acid and bile salts [8].

In recent years, many researchers have investigated the effects and mechanisms of various substances on bile salt resistance. Specifically, treatment of probiotics with exogenous substances, such as lactose [9], soy lecithin [10], whey protein isolates [11], maltodextrin [12], and lotus seed resistant starch [13], can effectively enhance bile salt tolerance by enhancing cell hydrophobicity, altering the fatty acid composition of the cell membranes, and inducing the expression of bile salt hydrolase genes. However, the initial survival rate of probiotics cannot be recovered after treatment with these substances, and different effects are observed in different species. Bile salts damage bacterial cell membranes by altering the composition of membrane lipids through changing the production of proteins involved in fatty acid metabolism [14-16]. They also cause cell death by disrupting the lipid packaging and proton motive forces [17]. Additionally, they cause DNA and RNA oxidative damage, protein misfolding [18], and intracellular acidification [19]. Therefore, the addition of a single exogenous substance may not be sufficient to prevent the degradation of probiotics. Hence, we speculated that a simultaneous treatment of probiotics with different substances may further improve the bile salt tolerance.

Whey protein isolates not only enhanced bile salt tolerance but also improved acid tolerance [11]. WPC 80, which contains more than 80% protein, is produced by removing a certain percentage of non-protein constituents from pasteurized whey derived from cheese processing. As a dry dairy ingredient, WPC 80 is generally used in food products and is more cost effective than whey protein isolate. Moreover, whey supplements can significantly alter the ratio of the range of proteins and fatty acids [20] and can act as a probiotic carrier for gastrointestinal transit [21]. Soybean lecithin, which is a byproduct of soybean oil processing and is composed of choline, fatty acids, glycerol, glycolipids, phospholipids, phosphoric acid and triglycerides, can enhance cell surface hydrophobicity and alter fatty acid composition to improve bile salt resistance [10]. To date, there has been no research studying the potential of combining the different substances mentioned above. Therefore, in our study, we designed a novel method that combines soybean lecithin and WPC 80 to treat *L. paracasei* L9 and assessed its effects on bile salt tolerance enhancement.

## Materials and Methods

### Organisms, Media and Growth Conditions

The strain *L. paracasei* L9 was provided by China Agricultural University. *Streptococcus thermophiles* G1, *Lactobacillus bulgaricus* L1 and *Lactobacillus rhamnosus* H024-A-15 were selected from fermented foods from Dali, China. *L. paracasei* L9, *L. bulgaricus* L1 and *L. rhamnosus* H024-A-15 were cultured in DeMan, Rogosa and Sharpe (MRS) medium at 38°C under anaerobic conditions. *S. thermophiles* G1 was cultured in M17 broth at 38°C under anaerobic conditions.

The bile salt-MRS/M17 (BS-MRS/M17) medium used to test the bile salt tolerance was prepared by adding different concentrations ((w/v)%) of cow bile salt (Gentihold, China) to MRS/M17 broth, buffered with 0.1 mol/L sodium phosphate to a final pH of 7.3, and sterilized at 121°C for 20 min.

The soybean lecithin-MRS/M17 (SL-MRS/M17) medium was prepared by adding different concentrations ((w/v)%) of soybean lecithin (Beijing Land Bridge, China) to MRS/M17 and sterilizing it at 121°C for 20 min after adjusting the pH to 6.4.

The WPC 80-MRS/M17 medium was prepared by adding different concentrations ((w/v)%) of WPC 80 (Friesland Campina DMV, The Netherlands), whose concentration was twice the final concentration, filtered with a 0.22 μm polyethersulfone (PES) filter, and mixed with an equal volume of double-strength MRS/M17, which was sterilized at 121°C for 20 min with the final pH adjusted to 6.4.

The WPC 80-SL-MRS/M17 broth was prepared by adding different concentrations of WPC 80, whose concentration was twice the final concentration, filtered with a 0.22 μm PES filter, and mixed with an equal volume of different concentrations of SL-MRS/M17. The concentration of each substance was twice the final concentration and the medium was sterilized at 121°C for 20 min with the final pH adjusted to 6.4.

### Bile Salt Tolerance

The bile salt tolerance was assessed as previously described by Hu *et al*. [22], with slight modifications. First, *L. paracasei* L9, *L. bulgaricus* L1 and *L. rhamnosus* H024-A-15 were aerobically cultured in MRS, WPC 80-MRS, SL-MRS, and WPC 80-SL-MRS broths with 2% (v/v) inocula at 38°C for 18 h, while *S. thermophiles* G1 was aerobically cultured in M17, WPC 80-M17, SL-M17, and WPC 80-SL-M17 broths with 2% (v/v) inocula at 38°C for 18 h. Following this, 1 ml samples of the fermentation broths were acquired, centrifuged at 2,235 g for 10 min to sediment a pellet, and then resuspended homogeneously in 1 mL BS-MRS/M17 medium with 0.3% (w/v) cow bile salt. The control groups were mixed evenly with MRS/M17 without cow bile salt. The mixtures were aerobically incubated at 38°C for 2.5 h. After that, the mixtures were centrifuged at 2,235 g for 10 min, the supernatants were discarded, and the pellets were serially diluted in normal saline. The viable cell counts were enumerated by pour plating using MRS/M17 agar and aerobic incubation at 38°C for 48 h, and the procedure was triplicated. The bile salt tolerance was expressed as the survival rate according to the following equation:

$$\text{Survival rate (\%) = }\frac{C_{1}}{C_{0}}\times 100\%,$$

where C_0_ is the viable cell counts in the culture medium before the cow bile salt challenge, and C_1_ is the viable cell counts in the culture medium after the cow bile salt challenge, respectively.

### Acid Tolerance

Acid tolerance was assessed as previously described [11] with slight modifications. *L. paracasei* L9, *L. bulgaricus* L1 and *L. rhamnosus* H024-A-15 were aerobically cultured in MRS, WPC 80-MRS, SL-MRS, and WPC 80-SL-MRS broths with 2% (v/v) inocula at 38°C for 18 h. *S. thermophiles* G1 was aerobically cultured in M17, WPC 80-M17, SL-M17, and WPC 80-SL-M17 broths with 2% (v/v) inocula at 38°C for 18 h. Subsequently, 1 ml samples of the fermentation broths were acquired, centrifuged at 2,235 g for 10 min, and the supernatants were discarded. The pellets were resuspended in 1 ml of MRS/M17 broth with the final pH adjusted to 2.0, and the control groups were mixed evenly in MRS/M17 broth. The cell suspensions were aerobically incubated at 38°C for 2 h. Then, the mixtures were centrifuged at 2,235 g for 10 min, the supernatants was discarded, and the precipitates were serially diluted with normal saline. The viable cell counts were enumerated by pour plating using MRS/M17 agar and aerobic incubation at 38°C for 48 h. The procedure was triplicated. The acid tolerance was expressed as the survival rate according to the following equation:

$$\text{Survival rate (\%) = }\frac{C_{1}}{C_{0}}\times 100\%,$$

where C_0_ is the viable cell counts in the culture medium before the acid challenge, and C_1_ is the viable cell counts in the culture medium after the acid challenge, respectively.

### Central Composite Design and Statistical Analysis

The experimental designs for response surface methodology (RSM), regression analysis and variance analysis were performed using Design Expert 8.0.6 (Stat-Ease, Inc., USA). Statistical analyses were performed using a two-way analysis of variance (ANOVA) with the GraphPad Prism software (GraphPad Software, Inc., USA). All experiments were conducted in triplicate and the results provided as mean ± SD. Statistical significance was set at *p* < 0.05.

## Results

### Bile Salt Tolerance of *L. paracasei* L9

**Fig. 1:**
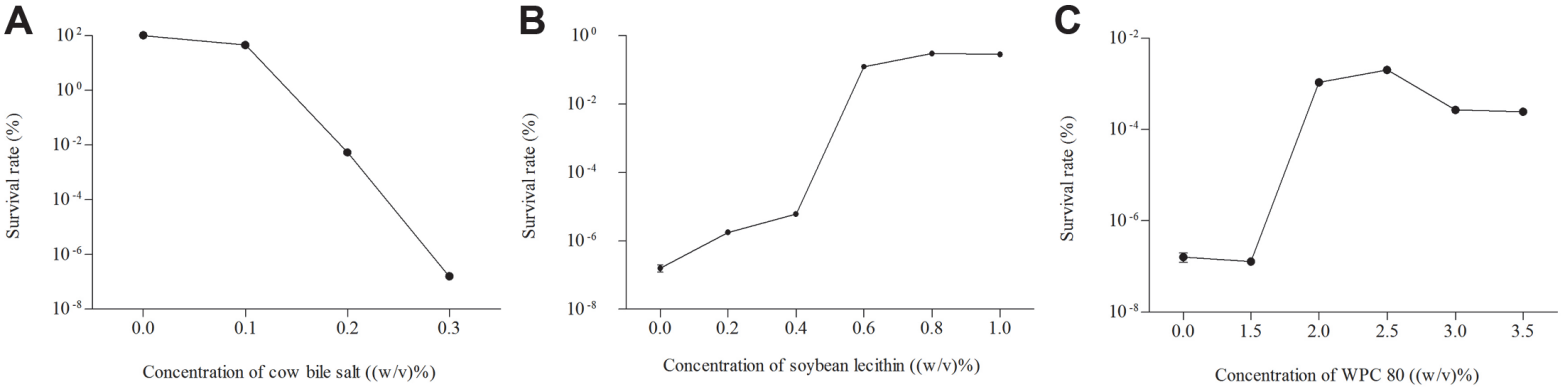


First, we characterized the bile salt tolerance of *L. paracasei* L9 via the method as described above, and we observed that the survival rate sharply decreased with increasing cow bile salt concentration (Fig. 1A). The survival rate decreased to 44.9 ± 4.6% and 0.005 ± 0.0007% when the bile salt concentration was 0.1% and 0.2%, respectively. Ultimately, after incubation for 2.5 h in BS-MRS broth with 0.3% cow bile salt, the survival rate nearly reduced to 0, in correspondence to the viable cell counts less than 100 CFU/ml. These results conclusively indicate that *L. paracasei* L9 is sensitive to bile salt.

### Effect of Soybean Lecithin on the Bile Salt Tolerance of *L. paracasei* L9

We subsequently assessed the effects of different concentrations (0.2, 0.4, 0.6, 0.8, 1.0% (w/v)) of soybean lecithin on the bile salt tolerance of *L. paracasei* L9, which was incubated with BS-MRS for 2.5 h (Fig. 1B). Soybean lecithin demonstrated no obvious effect on cell density, but it significantly increased the survival rate of BS-MRS-treated *L. paracasei* L9. Specifically, the survival rate continued to increase with increasing concentration of soybean lecithin in the range of 0.4%-0.8% (w/v). The survival rate remained stable at 0.28%-0.30% when the concentration was more than 0.8% (w/v). In summary, even though the survival rate of *L. paracasei* L9 stayed at a low level after treatment with soybean lecithin, it still significantly improved the bile salt tolerance of *L. paracasei* L9 within a certain concentration range.

### Effect of WPC 80 on the Bile Salt Tolerance of *L. paracasei* L9

We next investigated the effect of WPC 80 on the bile salt tolerance of *L. paracasei* L9 over 2.5 h of incubation in BS-MRS (Fig. 1C). Generally, WPC 80 showed a positive effect, especially at concentration of 2.5%, not only on cell density but also on the survival rate. Even so, the survival rate of *L. paracasei* L9 still improved after WPC 80 treatment. The survival rate increased initially and then subsequently dropped slightly with increasing WPC 80 concentration. The survival rate peaked (0.002 ± 0.0001%) after treatment with 2.5% (w/v) WPC 80, and then slightly decreased when the concentration of WPC 80 was higher than 2.5% (w/v). Together, the results indicate that WPC 80 positively enhanced the bile salt tolerance (survival rate increased almost 1 × 10^4^ times) of *L. paracasei* L9, even though the survival rate stayed at a low level.

### Experimental Design and Results of Central Composite Design

Although soybean lecithin and WPC 80 demonstrated significant effects on the bile salt tolerance of *L. paracasei* L9, the survival rate still stayed at an extremely low level after treatment with 0.3% (w/v) cow bile salt. Therefore, we designed an RSM to verify the assumption that the interactions of the two materials would further enhance the bile salt tolerance of *L. paracasei* L9. The experimental design and results of the central composite design are shown in Table 1.

### Regression Analysis

Based on the central composite design results, we obtained the quadratic regression model using Design Export 8.0.6. The regression function with the two variables can be expressed as:

R = −3.94 + 13.74A + 6.56B + 1.99AB − 12.74A^2^ − 1.58B^2^,

where R, A, and B represent log10 viable cell counts, soybean lecithin concentration, and WPC 80 concentration, respectively.

### ANOVA for the Response Surface Quadratic Model

Next, we estimated the validation of the model based on statistical significance by performing an ANOVA. ANOVA for the regression equation of log10 viable cell counts is shown in Table 2. The results indicate that the model is extremely significant, and there is a slight chance that a large “Model F-value” can occur due to noise. All model terms, including A, B, AB, A^2^, and B^2^ significantly contributed to the response value R (pA < 0.01, pB < 0.01, pAB < 0.01, pA^2^ < 0.01, pB^2^ < 0.01). Meanwhile, the lack of fit (*p* > 0.05) was not significant. Additionally, the fit statistics for the regression equation (Table 3) indicated that the predicted equation of the model could explain 99.63% of the variability in the log10 viable counts of *L. paracasei* L9. In summary, the quadratic equation model could effectively describe the relationship between soybean lecithin and WPC 80 for the log10 viable cell counts of *L. paracasei* L9 in 0.3% (w/v) cow bile salt.

**Fig. 2:**
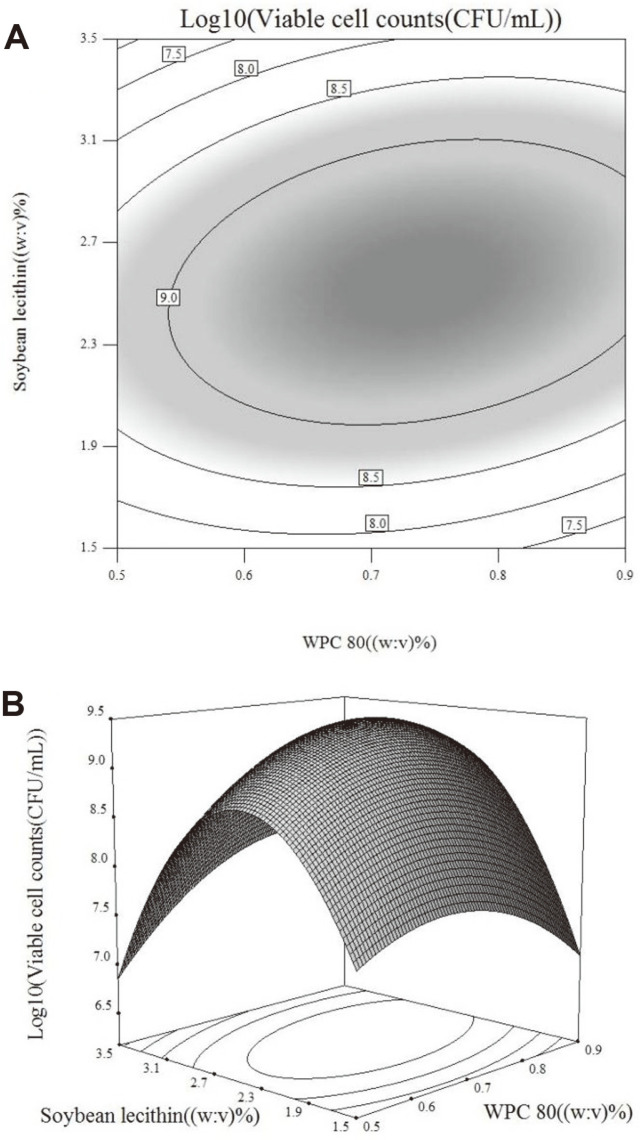


Furthermore, to estimate the effect of the interaction of soybean lecithin and WPC 80 on the response variable, we constructed two-dimensional contour and three-dimensional plots (Fig. 2) of the response against soybean lecithin and WPC 80. The two plots demonstrated the variation in the log10 viable cell counts with various concentrations of soybean lecithin and WPC 80, and it appears to have a single optimum condition. Meanwhile, the elliptical contour plots indicated that the interaction between soybean lecithin and WPC 80 (AB) was significantly important (pAB < 0.01) for the log10 viable cell counts.

**Fig. 3:**
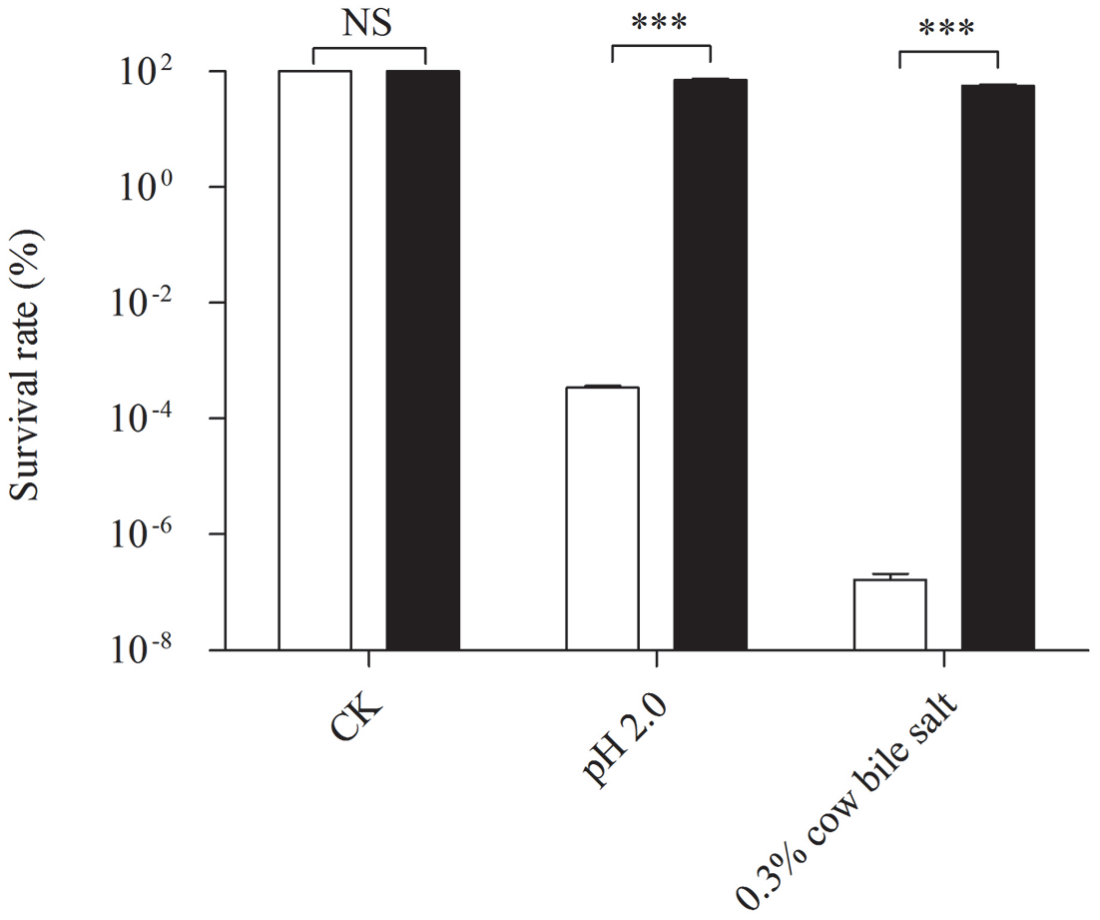


According to the quadratic function, we ascertained the estimated maximum response value of log10 viable cell counts 9.46 corresponding to viable cell counts of 2.89 × 10^9^ CFU/ml at optimal settings of 0.74% soybean lecithin and 2.54% WPC 80. Then, we performed a confirmation experiment under the estimated optimal settings to evaluate the accuracy of the quadratic model. The results (Fig. 3) showed that the observed response value of viable cell counts was 2.97 × 10^9^ CFU/ml, corresponding to log10 viable cell counts of 9.47 (*n* = 3). There was no significant difference between the estimated value and observed value. The results illustrated the suitability of the model to reflect the relationship of the log10 viable cell counts with soybean lecithin and WPC 80 concentrations. After optimization, the loss of log10 viable cell counts of *L. paracasei* L9 decreased to lower than 0.2. Correspondingly, the survival rate increased to 52.5 ± 4.7%. Conclusively, the combination of soybean lecithin and WPC 80 could improve the bile salt tolerance more effectively than the methods used before optimization.

Due to the acquisition of bile salt resistance could increase the survival rate of *Bifidobacterium* under low-pH conditions [23], we estimated the accessibility of the optimized method for *L. paracasei* L9 against low pH condition. We found that the survival rate of *L. paracasei* L9 was increased to 71.25% after inoculation in WPC 80-SL-MRS at a ratio of 0.74% soybean lecithin and 2.54% WPC 80, compared to the survival rate of 0.0003% of control group (Fig. 3).

**Fig. 4:**
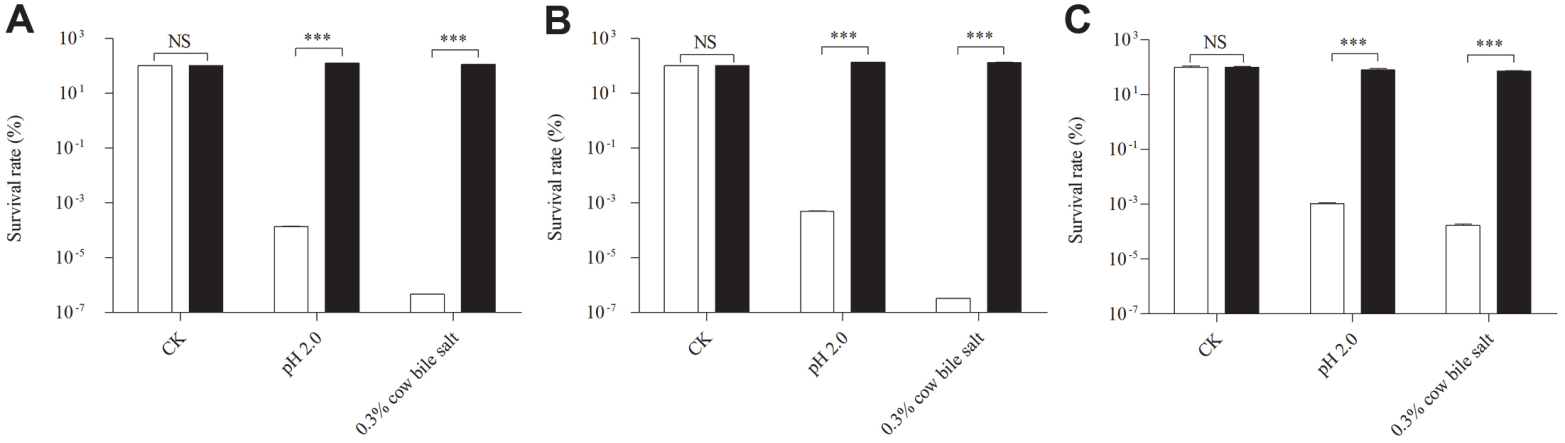


Additionally, we also tested the general applicability of the optimized method for other LAB. The results illustrated that the effectiveness of this method was also applicable for other probiotics, such as *S. thermophiles* G1, *L. bulgaricus* L1, and *L. rhamnosus* H024-A-15 (Fig. 4). Interestingly, more than 100% survival rates of *S. thermophiles* G1 and *L. bulgaricus* L1 are acquired in 0.3% (w/v) cow bile salt and pH 2.0 conditions after inoculation with the optimized method, but that was not observed in *L. paracasei* L9 and *L. rhamnosus* H024-A-15. The phenomenon may due to the propagation of the strains in MRS medium when soybean lecithin and WPC 80 constructed a non-lethal environment against bile salt and low pH conditions. All in all, the combination of soybean lecithin and WPC 80 at a special ratio could effectively protect probiotics against gastrointestinal stresses.

## Discussion

*L. paracasei* L9 is characterized by high lactic acid production and various health-promoting functions, such as regulating host immunity, modulating human gut microflora, and preventing allergic sensitization [24] and particulate matter exposure [25]. However, the bile salt tolerance of *L. paracasei* L9 has not been studied to date. Therefore, we investigated the bile salt tolerance of *L. paracasei* L9 and determined that it is a bile salt-sensitive strain. Generally, strategies to improve the bile salt tolerance of probiotics include isolation and screening from the natural environment [26], addition of exogenous substances [9-11], mutation breeding, acclimatization [23], gene modification by genetic engineering, metabolism engineering [27, 28], and microencapsulation technologies [29, 30]. However, it is difficult to isolate and screen probiotic strains with both health benefits and bile salt tolerance in nature. The methods of mutation breeding and acclimatization are indeterminate and non-oriented. The genetically modified strains with excess production of bile salt hydrolase not only enhance the bile salt tolerance but also inhibit cell growth due to bile acid micelles in the cytoplasm [31]. Additionally, the joint FAO/WHO definition of probiotics excluded genetically modified organisms that were applied to food. Although, microencapsulation permits cell maintenance and growth, the materials used for encapsulation lack cell-recognition sites and may yield toxic degradation products that can cause unpredictable inflammation [32]. The previously reported microencapsulation techniques are also limited by various factors, such as low viability and activity of probiotics, high energy consumption, insufficient protection against stress conditions, and difficulty in scaling up production [33].

Some studies demonstrated that whey protein can improve the resistance to bile salts in *Streptococcus thermophiles* ST-M5 and *Lactobacillus bulgaricus* LB-12 by slowing down the damage of proteins or facilitating protein repair [11]. Soybean lecithin can enhance the cell surface hydrophobicity and membrane integrity of *Lactobacillus plantarum* by altering the fatty acid composition [10]. The addition of soy protein can bind bile acids, aggregating them to partially alleviate the inhibition of *Bifidobacterium breve* Yakult by bile [34]. Additionally, lactose can also enhance the bile salt tolerance of *Lactobacillus bulgaricus* and *Streptococcus thermophiles* [9] probably because it provides galactose to form hexasaccharide-phosphate repeating units in the cell wall [35]. Even though the effects of these methods were limited, they did work by alleviating the cell membrane damage and altering the fatty acid composition. Due to the complexity of destruction caused by bile salts and acids, we hypothesized that combination of different components, especially proteins and fatty acids, could remedy the deficiency of previous studies so as to enhance the tolerance of probiotics against gastrointestinal stresses by constructing a shell to neutralize the destructions from bile salt and acid. Here, our studies indicate that combination of soybean lecithin and WPC 80 does enhance survival rates of probiotics in bile salt and acid conditions significantly. Our method improves on previous studies and is also easy to apply in large-scale productions and is generally applicable to other strains. Overall, this study provides a strategy against environmental stresses by mimicking the cell wall and cell membrane of lactic acid bacteria.

## Figures and Tables

**Table 1:** Design and results of central composite design for response surface methodology.

Run	Factor A Soybean lecithin ((w/v)%)	Factor B WPC 80 ((w/v)%)	Response Log_10_(viable cell count (CFU/ml))
1	0.60	2.50	9.23735
2	0.60	2.50	9.22011
3	0.60	2.50	9.27184
4	0.50	3.00	8.18136
5	0.60	2.50	9.22011
6	0.60	1.79	8.54218
7	0.70	2.00	9.05098
8	0.60	3.21	8.36326
9	0.46	2.50	8.54195
10	0.70	3.00	9.11844
11	0.60	2.50	9.24597
12	0.74	2.50	9.4302
13	0.50	2.00	8.51178

**Table 2:** ANOVA analysis for regression equation.

Source	Sum of squares	df	Mean square	F Value	*p*-value Prob>F
Model	2.13	5	0.43	375.12	< 0.0001^***^
A	0.07	1	0.07	61.42	0.0001^***^
B	0.46	1	0.46	403.54	< 0.0001^***^
AB	0.04	1	0.04	34.89	0.0006^***^
A^2^	0.11	1	0.11	99.6	< 0.0001^***^
B^2^	1.08	1	1.08	952.56	< 0.0001^***^
Residual	7.94E-03	7	1.13E-03		
Lack of Fit	6.10E-03	3	2.03E-03	4.41	0.0929
Pure Error	1.84E-03	4	4.61E-04		
Cor Total	2.14	12			

^a^ ***means *p* < 0.001.

**Table 3:** Fit statistics for regression equation.

Source	Value	Source	Value
Standard deviation	0.034	R-Squared	0.9963
Mean	8.92	Adjusted R-Squared	0.9936
C.V. %	0.38	Predicted R-Squared	0.9783
PRESS	0.046	Adequate Precision	54.859
